# The impact of SARS-CoV-2 on emergency health care in a referral acute-care center in northern Italy

**DOI:** 10.1093/eurpub/ckac129.540

**Published:** 2022-10-25

**Authors:** A Ferrari, F Parente, G Iudica, M Porretto, D Simonetta, C Minet, S Mosca, D Panatto, A Orsi, G Icardi

**Affiliations:** 1 Department of Health Sciences, University of Genoa, Genoa, Italy; 2 Interuniversity Center on Influenza and Other Transmissible Infections, Genoa, Italy; 3 Ospedale Policlinico San Martino IRCCS, Genoa, Italy

## Abstract

To assess how SARS-CoV-2 has changed the demand for in-person health care, we retrospectively analyzed data on access to the emergency department (ED) of San Martino Hospital, the referral acute-care center in the Liguria region (Northwest Italy). 181,699 records of patients diagnosed with an ICD-9 code between 2019 and 2021 were considered. In comparison to pre-pandemic levels, following the introduction of social distancing measures, the median number of ED visits declined by 41.4% in 2020 and by 28.1% in 2021. The period of maximum drop in access (-58.6%) corresponded to the 2020 11-12th calendar weeks and coincided with the highest rates of COVID-like illness - defined as either ILI or LRTI cases - identified through an operator-dependent syndromic surveillance system (+340%; 19.5% of total ED attendances). In terms of relative impact, in 2020 and 2021 non-urgent ED codes decreased (by 6.7% and 7.3%) and both urgent and emergency ED codes increased (by 4.8% and 3.8% the former; 5.5% and 8.8% the latter), even so, the absolute number of ED access fell drastically for all codes. Urgent codes, in particular, experienced the most severe decrease, shifting from a pre-pandemic value of 25,009 to 18,826 in 2020 and 19,528 in 2021. With regards to diagnosis, in 2020, respiratory infections saw the highest increase (+3.3%) while traumas and eye diseases saw the highest decrease (-1.1% and -3.8%, respectively). This trend reversed in 2021 during which respiratory infections decreased (-2.2%) and traumas increased (+2.2%). Despite the admissions of males and the elderly being routinely lower, these categories experienced the greatest increase in access for respiratory infections: +3.9% and +10.1% in 2020; +2.8% and +7.4% in 2021. While reduction of non-urgent ED visits indicates that the high pre-pandemic access levels may have been avoidable, the significant decline in non-COVID-19 urgent accesses potentially points to an increase in delayed and missed care.

**Key messages:**

• During the COVID-19 pandemic – possibly due to fear and underestimation of symptoms – there was an overall reduction in ED accesses that potentially points to an increase in delayed or missed care.

• The reduction in non-urgent attendances indicates that high pre-pandemic accesses may have been avoidable and that a reduction in unnecessary ED visits is an attainable goal for healthcare systems.

